# Macrophage Activation Syndrome as Onset of Systemic Lupus Erythematosus: A Case Report and a Review of the Literature

**DOI:** 10.1155/2015/294041

**Published:** 2015-05-07

**Authors:** Guido Granata, Dario Didona, Giuseppina Stifano, Aldo Feola, Massimo Granata

**Affiliations:** ^1^UOC Immunologia Clinica A, Dipartimento di Medicina Clinica, Policlinico Umberto I, Sapienza Università di Roma, 00185 Roma, Italy; ^2^I Divisione Dermatologica, Istituto Dermopatico dell'Immacolata IRCCS, 00167 Roma, Italy

## Abstract

Macrophage activation syndrome (MAS) is a potentially fatal condition. It belongs to the hemophagocytic lymphohistiocytosis group of diseases. In adults, MAS is rarely associated with systemic lupus erythematosus, but it also arises as complication of several systemic autoimmune disorders, like ankylosing spondylitis, rheumatoid arthritis, and adult-onset Still's disease. Several treatment options for MAS have been reported in the literature, including a therapeutic regimen of etoposide, dexamethasone, and cyclosporine. Here we report a case of 42-year-old woman in whom MAS occurred as onset of systemic lupus erythematosus.

## 1. Introduction

Macrophage activation syndrome (MAS) is a potentially fatal condition. It is a rare complication of several autoimmune disorders, including systemic lupus erythematosus (SLE) and systemic juvenile idiopathic arthritis (sJIA). The incidence of MAS associated with SLE is about 0.9–4.6% [1]. MAS is a multifarious disease, presenting with several signs and symptoms, including high fever, hepatomegaly, splenomegaly, hemorrhagic manifestations (e.g., purpura), and dysfunction of the central nervous system, like lethargy. Furthermore, MAS is characterized by several alterations in laboratory tests, including pancytopenia, hypofibrinogenemia, hypertriglyceridemia, and hyperferritinemia.

MAS is classified among the group of hemophagocytic lymphohistiocytosis (HLH), which includes familial HLH and secondary HLH. Secondary HLH is triggered by several causes, including infection, drugs, malignancy, and rheumatic disorder [2].

We report a rare case of MAS that occurred as first manifestation of SLE treated with high dose intravenous methylprednisolone and oral cyclosporine.

## 2. Report of Case

A previously healthy 42-year-old Caucasian woman was admitted to our department presenting an 8-week history of persistent fever up to 39°C with shiver unresponsive to antipyretics, dyspnea, weight loss, malaise, and lethargy. Her medical past history was unremarkable for rheumatic diseases, severe infections, or immunodeficiency. Her family history also was negative for rheumatic diseases.

Our clinical examination showed lymphadenopathy in the axillae, a widespread rash prominent on her lower legs, symmetric arthritis involving hands and wrists, and bilateral pulmonary basal crackles.

We started instrumental and laboratory tests to rule out the presence of autoimmune, infectious, or neoplastic disease. Repeated blood and urine cultures and a thorough infection screen including a viral panel for herpes zoster, herpes simplex (HSV-1, HSV-2), Epstein-Barr virus (EBV), cytomegalovirus, hepatitis B and C, HIV, coxsackie, and parvovirus B19 viruses were negative. The tuberculin sensitivity test (PPD test) was negative.

Laboratory routine showed pancytopenia, hypergammaglobulinemia, hyperferritinemia (1700 mg/dL), hypofibrinogenemia (100 mg/dL), hypertriglyceridemia (300 mg/dL), and raised levels of alanine aminotransferase (ALT), aspartate aminotransferase (AST), *γ*-glutamyl transferase, lactate dehydrogenase, and serum creatinine. The levels of blood urea nitrogen and total bilirubin were within reference range. We also repeated ESR every five days, founding a progressive reduction in levels, ranging from 120 mm/h to 2 mm/h.

Immunological screening was positive for ANA (1 : 240 homogenous), anti-dsDNA (40 UI/mL), anti-Sm (154.56 UI/mL), and anti-RNP (154.21 UI/m). Serum C3 and C4 complement factors were low, respectively, 0.43 g/L (range 0.65–1.65 g/L) and 0.07 g/L (range 0.16–0.6 g/L).

We performed an abdominal ultrasound exam, founding a moderate hepatosplenomegaly. Echocardiography revealed a diffuse pericardial effusion without valvular vegetations, oscillating intracardiac mass, abscess, or valvular regurgitation. Chest CT examination showed a right-basal parenchymal thickening, a bilateral pleural effusion, and multiple mediastinal and axillary lymphadenopathy. Bronchoalveolar lavage revealed neutrophils and bronchial cells smears with squamous metaplasia but excluded the presence of neoplastic cells. Total lymphocyte count and CD4^+^/CD8^+^ T lymphocyte ratio on bronchoalveolar lavage ruled out sarcoidosis. We also performed an axillary lymph node biopsy, which was negative for malignant lymphoproliferative disorder, and a bone marrow biopsy, which detected hemophagocytosis. The presence of pancytopenia, polyarthritis, pleural and pericardial effusion, and positive ANA, anti-dsDNA, and anti-Sm suggested a diagnosis of LES, according to the Systemic Lupus International Collaborating Clinics (SLICC) classification criteria [3]. At the same time, the particular evolution of the ESR in association with the evidence of hemophagocytosis in the bone marrow and the exclusion of infection led us to the main diagnosis of MAS, according to the hemophagocytic lymphohistiocytosis (HLH) criteria as follows [2].

HLH  criteria  (at  least  5  criteria  should  be  met  for  MAS  diagnosis)

 temperature ≥38.5°C for 7 days at least,
 spleen enlargement,
 hypertriglyceridemia (>160 mg/dL),
 hypofibrinogenemia (<150 mg/dL),
 ferritin ≥500 *μ*g/L,
 hepatitis,
 sIL-2 receptor >2400 IU/mL,
 decreased or absent NK cell activity,
 hemophagocytic cells in bone marrow, spleen, or lymph nodes,
 cytopenia in 2 or more cell lines (hemoglobin <9 g/dL, platelets <100 000/*μ*L, or neutrophils <1000/*μ*L).

We started high-dose intravenous methylprednisolone (1 g/day) for three days. Then the drug was switched to oral prednisolone 60 mg and cyclosporine 3 mg/kg daily. After the end of methylprednisolone therapy, the patient was apyretic; subsequently skin rash disappeared. The laboratory parameters returned to normal levels within 2 weeks. The patient was discharged from the hospital and is now under follow-up at the outpatient clinic.

## 3. Discussion

MAS belongs to the group of hemophagocytic lymphohistiocytosis (HLH), which includes familial HLH and secondary HLH. The association between HLH and autoimmune or autoinflammatory disease is formally called MAS [4]. MAS is commonly associated with sJIA [4] but has also been reported in Kawasaki disease, adult-onset Still's disease, rheumatoid arthritis, Sjögren's syndrome, dermatomyositis, mixed connective tissue disease, systemic sclerosis, and SLE [5]. MAS associated with SLE is rare and the incidence is about 0.9–4.6% [1]. To date, only 26 cases of MAS only related to the onset of SLE are reported in the literature [5–8]. We find other several cases of MAS due to SLE in the literature, but these cases are related to SLE flare-up or complication [5, 9].

The diagnosis of MAS is a challenge. Indeed, the clinical features of MAS-associated SLE and active SLE are very similar. In a recent paper it is reported that respiratory symptoms, jaundice, and lymphadenopathy are present in 75% of patients [10]. However, hyperferritinemia is reputed the best parameter to discriminate between MAS-associated SLE and active SLE with a sensitivity and specificity of almost 100% [1]. Nevertheless, severe leukopenia leads the clinician to evaluate the presence of MAS [11]. In our patient, the diagnosis of MAS-associated SLE was made according to HLH-2004 criteria (see HLH criteria) [2].

To date, the pathogenesis of MAS is not totally known. It is considered an intensive systemic inflammatory reaction, caused by a massive dysregulation of macrophage–lymphocyte interactions, which provokes increases in the levels of several cytokines, particularly TNF-*α*, M-CSF receptors, interleukin- (IL-) 1, IL-6, and interferon gamma- (IFN-) *γ* [12].

Index of suspicion for MAS is higher when an infection is ruled out or inflammation persists and does not respond to treatment of an underlying infection. In this case, it could be useful to start an immunomodulatory therapy even in the face of infection. To date, several therapeutic options are available, including nonbiologic and biologic treatments. The cornerstone is represented by steroids. An intravenous methylprednisolone pulse therapy (e.g., 30 mg/kg for three consecutive days) followed by 2-3 mg/kg per day is the most common schedule [2]. However, several drugs are used alone or in combination to reduce steroids. Parenteral administration of cyclosporine A (CSA) (2–7 mg/kg/day) is usually started if methylprednisolone lacks improvement [13]. In addition, CSA is used after the discharge of patients to maintain MAS under control [1]. In patients unresponsive to steroids and CSA, HLH-2004 treatment protocol could be useful [5]. This protocol includes dexamethasone, CSA, and etoposide. However, the potential liver toxicity of etoposide restricts the use of this therapeutic option. Recently, Coca et al. reported the use of antithymocyte globulin (ATG) as an alternative to etoposide [14], but infusion reactions to ATG are commonly described in the literature [15]. Although some authors reported the development of HLH after administration of TNF-*α* antagonist [16, 17], infliximab could be a useful treatment in refractory cases of MAS [18]. Rituximab [19] and intravenous immunoglobulin (IVIG) [20] have been employed with good results in MAS triggered by viruses, such as EBV or cytomegalovirus. In the pediatric community, IL-1 blockers had become almost standard of care for treatment of MAS regardless of underlying autoimmune disorder [21–25], although the role of IL-1 in the pathogenesis of MAS is still only partially known. However, some authors reported some cases of MAS possibly triggered by anakinra [24, 25].
